# *SPECC1L* Mutations Are Not Common in Sporadic Cases of Opitz G/BBB Syndrome

**DOI:** 10.3390/genes13020252

**Published:** 2022-01-28

**Authors:** Chiara Migliore, Anna Vendramin, Shane McKee, Paolo Prontera, Francesca Faravelli, Rani Sachdev, Patricia Dias, Martina Mascaro, Danilo Licastro, Germana Meroni

**Affiliations:** 1 Department of Life Sciences, University of Trieste, 34127 Trieste, Italy; migliore.chiara81@gmail.com (C.M.); martina.mascaro@phd.units.it (M.M.); 2 Genomic and Bioinformatic Lab., Cluster in Biomedicine, S.c.r.l., 34149 Trieste, Italy; anna.vendramin@gmail.com; 3 Northern Ireland Regional Genetics Service, Belfast City Hospital, Belfast BT9 7AB, UK; shane.mckee@belfasttrust.hscni.net; 4 Medical Genetics Unit, University and Hospital of Perugia, 06129 Perugia, Italy; paolo.prontera@ospedale.perugia.it; 5 The North East Thames Regional Genetics Service, Great Ormond Street Hospital, London WC1N 3JH, UK; Francesca.Faravelli@gosh.nhs.uk; 6 St George and Sydney Children’s Hospital, Randwick, Sydney, NSW 2031, Australia; Rani.Sachdev@health.nsw.gov.au; 7 Serviço de Genética Médica, Hospital de Santa Maria, Centro Universitário Hospitalar Lisboa Norte, 1649-028 Lisbon, Portugal; patricia.dias@chln.min-saude.pt; 8 Area Science Park, 34149 Trieste, Italy; danilo.licastro@areasciencepark.it

**Keywords:** Opitz G/BBB Syndrome, *SPECC1L* gene, *MID1* gene, hypospadias

## Abstract

Opitz G/BBB syndrome (OS) is a rare genetic developmental condition characterized by congenital defects along the midline of the body. The main clinical signs are represented by hypertelorism, laryngo–tracheo–esophageal defects and hypospadias. The X-linked form of the disease is associated with mutations in the *MID1* gene located in Xp22 whereas mutations in the *SPECC1L* gene in 22q11 have been linked to few cases of the autosomal dominant form of this disorder, as well as to other genetic syndromes. In this study, we have undertaken a mutation screening of the *SPECC1L* gene in samples of sporadic OS cases in which mutations in the *MID1* gene were excluded. The heterozygous missense variants identified are already reported in variant databases raising the issue of their pathogenetic meaning. Recently, it was reported that some clinical manifestations peculiar to OS signs are not observed in patients carrying mutations in the *SPECC1L* gene, leading to the proposal of the designation of ‘*SPECC1L* syndrome’ to refer to this disorder. Our study confirms that patients with diagnosis of OS, mainly characterized by the presence of hypospadias and laryngo–tracheo–esophageal defects, do not carry pathogenic *SPECC1L* mutations. In addition, *SPECC1L* syndrome-associated mutations are clustered in two specific domains of the protein, whereas the missense variants detected in our work lies elsewhere and the impact of these variants in the function of this protein is difficult to ascertain with the current knowledge and will require further investigations. Nonetheless, our study provides further insight into the *SPECC1L* syndrome classification.

## 1. Introduction

Opitz G/BBB Syndrome (OS; OMIM 300000, 145410) is a multiple congenital anomaly disorder characterized by defects in the development of ventral midline structures and by variable expressivity of the clinical signs. Clinical manifestations of the disease comprise facial dysmorphic features, which include hypertelorism, prominent forehead, broad nasal bridge, low-set-posteriorly rotated ears, cleft lip and/or palate; laryngo–tracheo–esophageal abnormalities; urogenital anomalies, including hypospadias, cryptorchidism and hypoplastic/bifid scrotum [1,2]. Other malformations are less frequent and involve atrial and ventricular septal defects, patent ductus arteriosus, coarctation of the aorta, imperforate or ectopic anus. Further, neurological manifestations are also reported, both as anatomical brain abnormalities, such as Dandy–Walker malformation, agenesis or hypoplasia of the corpus callosum, and/or cerebellar vermis, as well as delayed development and intellectual disability [3].

OS is genetically heterogeneous, presenting with an X-linked (XLOS, OMIM 300000) and an autosomal dominant form (ADOS, OMIM 145410) [4]. The two forms were reported to be clinically indistinguishable and, interestingly even in the autosomal form; males are usually more severely affected than females [3]. XLOS is associated with pathological variants in the *MID1* gene located on the short arm of the X chromosome (Xp22.2) [5]. The *MID1* gene product is an E3 ubiquitin ligase associated with microtubules, involved in several cellular signaling pathways [6,7,8,9,10]. Both familial and sporadic pathogenic variants affecting *MID1* have been reported to date in approximately 90 OS male patients, accounting for approximately 20−30% of screened OS cases [5,7,11,12,13,14,15,16,17,18,19,20,21,22,23,24,25]. The distribution and type of mutations indicate that loss-of-function is the mechanism underlying the pathogenesis of XLOS [12].

Conversely, ADOS is linked to genomic losses in the 22q11.2 region [4]. Deletions in this same region may also result in DiGeorge syndrome (OMIM 188400) and velocardiofacial syndrome (OMIM 192430), which share some clinical signs with OS, but the genotype–phenotype correlation is not clear. More recently, Kruszka et al. reported two different pathogenic variants in the *SPECC1L* gene, located at chromosome 22q11.23, segregating with ADOS in a three-generation family, originally reported [4] and in an additional family with autosomal-dominant clinical manifestations, further supporting the ADOS linkage to the 22q11.2 region [26]. However, other reports indicate the involvement of *SPECC1L* mutations in other conditions only partially overlapping with OS, such as oblique facial cleft (ObFC, OMIM 600251), Teebi hypertelorism (THS, OMIM 145420) and Baraitser–Winter (BWS, OMIM 243310) syndromes [27]. Thus, the phenotypic spectrum associated with *SPECC1L* mutations is complex and still not completely unraveled.

Our report, addressing whether mutations in this gene can account for sporadic OS-diagnosed patients, provides further insights into the *SPECC1L* syndrome classification.

## 2. Patients and Methods

*Patients*. We molecularly investigated 30 unrelated sporadic patients—4 females and 26 males—diagnosed as OS cases in Italian and European genetics laboratories and all presenting normal karyotype. The main phenotypic features are reported in the next section.

*PCR amplification and Sanger sequencing*. Genomic DNA from the OS subjects was available for molecular studies and mutation screening of the *MID1* coding region was performed as previously reported [14]. Amplification products were sequenced with a BigDye Terminator Sequencing Kit in an ABI3730XL Genetic Analyzer (Life Technologies, Paisley, UK). PCR amplification of the *SPECC1L* gene was performed as above using primer sets for coding exons 3 to 17, designed based on previous report [26]. Sequences were analyzed by comparison with the human genome reference assembly (GRCh38/hg38) using the CodonCode Aligner software (version 3.7.1).

*Whole-Exome Sequencing (WES).* For the sample analyzed with WES, one microgram of genomic DNA was sheared by sonication using a Covaris M220 station. The library was prepared according to a TruSeq Exome Enrichment kit (Illumina, San Diego, CA, USA) targeting 62 Mb of the human genome (20.794 genes). Indexed exome-enriched libraries were qualitatively and quantitatively assessed with an Agilent 2100 Bioanalyzer and qPCR KAPA Library Quantification kit before and after the pooling for sequencing. Sequencing was carried out using an Illumina HiScanSQ Platform with a Truseq SBs kit V3 2 × 100 bp in pair-end mode (Illumina, CA, USA). High-quality reads were aligned to the human reference genome (GRCh37/hg19) following GATK recommendation.

*In silico analyses of SPECC1L missense variants*. Reported missense variants from Sanger were identified in the NCBI dbSNP (http://www.ncbi.nlm.nih.gov/SNP/ accessed on 30 November 2021) and GnomAD (http://gnomad.broadinstitute.org/ accessed on 30 November 2021) databases, and the available global and subset-specific frequencies were reported. Prediction of the effect of missense variants and the likelihood of pathogenicity was carried out using several open source tools for clinical interpretation of genetic variants as per the ACMG/AMP 2015 guidelines [28]. In particular, we reported scores from the following: MutationTaster (http://www.mutationtaster.org/ accessed on 30 November 2021) [29]; PolyPhen2 (http://genetics.bwh.harvard.edu/pph2/ accessed on 30 November 2021) [30]; and SIFT (https://sift.bii.a-star.edu.sg/ accessed on 30 November 2021) [31]. The position of variants relative to the *SPECC1L* protein domains was performed using NextProt (NEXT.PROT.org/*SPECC1L*/SEQUENCE) and the impact of the missense variants on the predicted coiled-coil domains and global protein structure was investigated with the ExPasy COILS (https://embnet.vital-it.ch/software/COILS_form.html, accessed on 3 December 2021) [32] and AlphaFold Protein Structure Database (https://alphafold.ebi.ac.uk/ accessed on 3 December 2021) [33], respectively.

## 3. Results

A cohort of 30 unrelated probands clinically referred as OS patients was analyzed in this study. Consistent with the most stringent criteria for diagnosis of OS [15], hypertelorism and hypospadias in males were the most common clinical signs reported. Almost 50% of the cases also displayed mild-to-severe developmental delay and intellectual disabilities, though only few of them were reported with assessed brain abnormalities. Cleft lip and palate and laryngo–tracheo–esophageal abnormalities were present in 40% of these sporadic cases. Heart defects, such as atrial and ventricular septal defects, patent ductus arteriosus and coarctation of the aorta, were diagnosed in 30% of patients and a small subset of individuals displayed anal abnormalities (Table 1).

**Figure 1:**
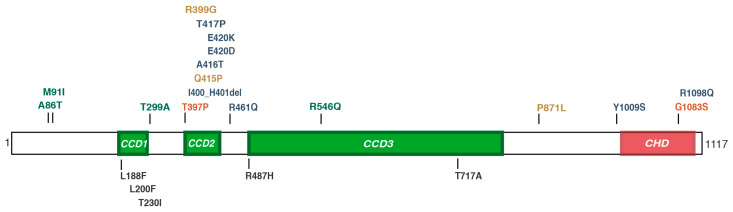
Schematic representation of the *SPECC1L* protein. Domain structure and positions of *SPECC1L* (Q69YQ0) are obtained from UniProt (www.uniprot.org, accessed on 30 November 2021). CCD1, coiled coil domain 1 (aa 160–280); CCD2, coiled coil domain 2 (aa 394–449); CCD3, coiled coil domain 3 (aa 487–807); CHD, calponin-homology domain (aa 1011–1116). The missense variants identified in our work are shown below the scheme. The mutations reported so far in literature are shown above the scheme with the following color-code: blue—found in Teebi syndrome patients; yellow—found in OFC, CDH and craniosynostosis patients; red—found in ADOS; green—found in non-syndromic orofacial cleft cases [26,27,34,35,36,37,38,39].

The presence of mutations in the coding and splice site regions of the *MID1* gene was excluded by direct amplification and Sanger sequencing of the nine *MID1* coding exons. One of these 30 samples was analyzed by whole-exome sequencing (WES, OS269) as this patient present with the two main OS clinical manifestation, i.e., hypertelorism and hypospadias. A missense variant in the *SPECC1L* gene was detected (see below) and we thus reasoned that DNA samples obtained from sporadic OS cases could carry heterozygous mutations in the *SPECC1L* gene as reported in some pedigrees of the autosomal form of OS [26]. These 30 samples were therefore directly screened by PCR and sequencing of the entire *SPECC1L* coding region (NM_015330.4). We identified five variants with minor allele frequency (MAF) <0.01 and missense outcome in six of these OS samples. The missense variants are listed in Table 2, along with their in silico analyses and predicted clinical significance, as reported in Methods. The missense variants are also represented in their corresponding domain position in the lower side of Figure 1. In the same figure we indicated the mutations already reported as pathogenic in dominant OS, Teebi hypertelorism syndrome, ObFC and other clinical manifestations [26,27,34,35] (Figure 1).

Below is the description of the heterozygous *SPECC1L* non-synonymous changes detected in the six OS cases, all reported in the dbSNP database (Table 2 and Table 3 and Figure 1).

A *SPECC1L* c.562C>T change, causing the Leu188Phe variation, is present in two Caucasian male patients (OS310 and OS325). OS310 presents with hypertelorism, micropenis and learning difficulties. OS325 diagnosis reports microcephaly, bilateral hearing loss, facial dysmorphic features, high arched palate, esophageal atresia, atrial septal defects, and vertebral schisis. The leucine residue involved in this variant is located in the first coiled coil domain of *SPECC1L* and is predicted from benign to possibly damaging, according to the different predictors (Table 2).

For patient OS296 detailed clinical description was not available. In this sample, we detected a missense variant in the third coding exon, c.600A>T leading to a Leu200Phe amino acid change. As for the Leu188Phe variant above, the leucine residue is located in the first coiled coil domain of the protein and is highly conserved among eukaryotes. However, pathogenicity prediction tools assign a benign/tolerated classification in accordance with a global MAF of approximately 0.01 (Table 2 and Table 3).

A c.689C>T change leading to a Thr230Ile amino acid substitution was detected in a Caucasian male patient (OS331) whose diagnosis reports hypertelorism, labioschisis, clynodactyly, hypospadias, hypoplasia of corpus callosum and developmental delay. When analyzed according to ethnic origin, the global MAF of 0.0058 for this variant is reported being 0.0288 in EAS population but not reported in European population (OS331) (Table 2 and Table 3). The amino acid change is predicted as benign/tolerated by the prediction tools interrogated (Table 2).

The c.1460G>A variant, leading to the Arg487His change, is present in a boy of Caucasian origin (OS269) born with several dysmorphic facial features (hypertelorism, bossed forehead, depressed nasal bridge, anteverted nares, thin upper lip, auricular pits), umbilical hernia, coarctation of the aorta and hypospadias. The Arg487His variation is located in the third coiled coil of *SPECC1L* and is predicted as benign-to-probably-damaging by the different prediction tools (Figure 1, Table 2 and Table 3).

Another variant identified in our study involves the same coiled-coil domain of the protein as above, CCD3 (Figure 1). Patient OS336, a boy born to non-consanguineous parents, presents with craniofacial dysmorphisms including hypertelorism and cleft palate, feeding difficulties, stridor, polydactyly, hypospadias and hypotonia. In this subject, a c.2149A>G leading to a Thr717Ala missense variation was detected: this change concerns a weakly conserved residue predicted thus benign/tolerated according to SIFT/Polyphen2/MutationTaster prediction tools. Nevertheless, the frequency of this variant is low in 1000 Genome Project, GO-ESP and ExAC (Table 2 and Table 3). This case was further reviewed and subsequently differentially diagnosed with Gomez–Lopez–Hernadez syndrome, as he presented with the typical phenotypic triad: cerebellum rhombencephalosynapsis, bilateral parietal or parietooccipital alopecia, and trigeminal dysfunction associated with corneal opacities. Unfortunately, the patient died and no further molecular investigation and assessment can be performed.

Using the in silico splice predictors NetGene2 (https://services.healthtech.dtu.dk/service.php?NetGene2-2.42, accessed on 30 November 2021) and BDGP (https://www.fruitfly.org/seq_tools/splice.html, accessed on 30 November 2021), we also excluded potential effects of this nucleotide substitutions on splicing of the *SPECC1L* transcript.

Thus, in this work we detected five non-synonymous *SPECC1L* variants with allele frequency in the range 0.009–0.0006 in six OS patients’ samples. The population frequencies of these variants, however, are too elevated and clearly exclude them from being causative of a rare autosomal dominant syndrome. This possibly suggests a minor pathogenetic involvement, if any, which is discussed below.

**Figure 2:**
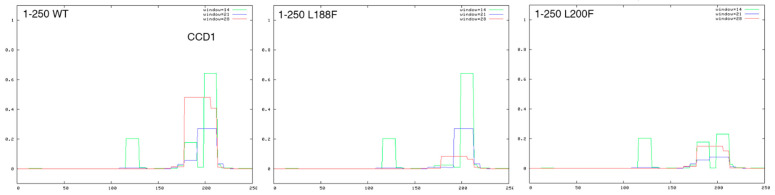
Missense mutations in the *SPECC1L* CCD1 domain. Coiled coil prediction of the first 250 residues of the *SPECC1L* WT (left box), Leu188Phe (middle box), and Leu200Phe (right box) sequence was performed with the ExPasy COILS tool (https://embnet.vital-it.ch/software/COILS_form.html, accessed on 3 December 2021). On the *X* axis is the aa position and, on the *Y* axis, the coiled-coil score (threshold 0.5). The color of the curves indicates the three window sizes used in the prediction (green, 14 aa; blue, 21 aa; red, 28 aa).

Interestingly, to date, ‘*SPECC1L* syndrome’ cases have been associated with mutations within two domains of the protein, the second coiled coil domain (CCD2) and the calponin-homology domain (CHD) [26,27,34,35,38,39] (Figure 1). On the contrary, missense variants residing outside, e.g., in coiled coil domains 3 (CCD3) or in other unstructured regions according to AlphaFold predictions, have been linked to non-syndromic orofacial clefting [37] and isolated craniosynostosis [36], raising the question of domain-dependent genotype–phenotype correlation. The variants we found in OS sporadic cases are located in CCD1 and CCD3 (Figure 1). Further analysis of the effect of two missense mutations in CCD1, using a coiled-coil predictor and alphaFold data, suggest a relevant role of these two leucine residues in the structure of CCD1. Regularly spaced leucine residues are important for coiled-coil structure and, accordingly, alphaFold predicts a ‘confident’ score for these two leucine residues within an α-helix composing CCD1 (not shown). Indeed, the score of the predicted coiled-coil region, principally based on the repetitive pattern of hydrophobic and hydrophilic residues, the heptad repeat, over three different window sizes (14, 21, and 28 residues), is reduced when the Leu188Phe substitution was plotted and almost abolished with the Leu200Phe substitutions (Figure 2). Thus, these leucine residues are important to determine CCD1 structure; unfortunately, the role of *SPECC1L* different domains is still not completely unraveled. It is tempting to speculate that regions outside CCD2 and CHD, where most of the assessed pathogenetic mutations are found, can contribute differently to *SPECC1L* function.

## 4. Discussion

Here, we report the molecular screening for mutations in the coding and splice site regions of the *SPECC1L* gene in samples of sporadic patients diagnosed with Opitz G/BBB syndrome who resulted negative for mutations in the *MID1* gene responsible for the X-linked form of the disease. The five non-synonymous variants identified are present in dbSNP with a MAF < 0.01 and, in some cases, well below that threshold; however, not so rarely as to be causative of a monogenic dominant syndrome.

The first pathogenic dominant mutations implicating the *SPECC1L* gene were reported in patients with oblique facial clefting (ObFC), a rare form of orofacial cleft [35]. An additional two mono-allelic pathogenic missense mutations were then reported in cases of autosomal dominant form of Opitz G/BBB syndrome, although mutations in this gene could not account for all chromosome 22q11.2-linked OS pedigrees [4,26]. Further mutations, a missense variant and an in-frame short deletion of 2 residues, were reported in patients diagnosed with an overlapping but distinct genetic disorders, Teebi hypertelorism syndrome [34]. More recently, Bhoj et al. reported six additional novel *SPECC1L* pathogenic variants in patients diagnosed with Teebi and Baraitser–Winter syndromes [27] (Figure 1). By thoroughly reviewing the spectrum of *SPECC1L* mutations-associated phenotypes, the authors concluded that, although the phenotypes observed in those patients overlap with OS signs, some clinical manifestations peculiar to OS are not observed in patients carrying mutations in the *SPECC1L* gene, i.e., hypospadias and laryngeal clefts, proposing the novel designation of ‘*SPECC1L* syndrome’ to refer to this disorder instead of autosomal dominant Opitz G/BBB Syndrome. An additional THS-associated mutation was reported, further expanding the *SPECC1L* phenotypic spectrum [27]. In addition, congenital diaphragmatic hernia (CDH) was reported as a prominent feature of *SPECC1L* syndrome [4]. Consistently, our study confirms that patients with diagnosis of OS, mainly characterized by the presence of hypospadias and absence of CDH, do not carry *SPECC1L* pathogenic missense variants, despite the fact that, in some cases, bioinformatic tools give contrasting significance predictions. Unfortunately, parents of the screened OS patients are not available for molecular assessment of the possible de novo status of the detected variants and the WES performed is, at present, non-conclusive. Only in one instance (p.Thr717Ala) dysmorphic features were also reported in the proband’s father suggesting, possible segregation of the genetic variant through the paternal family’s side.

The frequency of the variants identified in this study are mostly (four out of five) higher than 0.001, considered the maximum limit for disease-causing alleles in such rare dominant disorders [40]. The possible implication of *SPECC1L* mono-allelic variants in autosomal dominant Opitz G/BBB patients will, therefore, require further evaluation, especially if we consider the elevated number of rare missense substitutions detected in the *SPECC1L* gene. Indeed, GnomAD reports 479 missense variants showing allele frequency below 0.001 to as low as 3.977 × 10^−6^ in a total of 282,000 alleles, an unexpectedly high number of missense variants for a gene implicated in an autosomal dominant disorder. One can hypothesize that several of these rare variants may have mild phenotypic consequence, e.g., acting as modifiers in variable expressivity or being involved in more complex patterns of inheritance. In all, our study suggests a minor pathogenicity impact of the *SPECC1L* variants identified in OS cases, if any.

As presented above, it is possible that mutations/variants in regions of the protein outside CCD2 and CDH can have a less deleterious effect on the protein, leaving residual protein function in heterozygosity, or they can exert an effect in only some compartments during embryogenesis. This may result in reduced penetrance or expressivity with respect to the CCD2 and CDH mutations and thus be relevant as genetic background modifiers and/or be involved in more complex genetic inheritance. Unfortunately, at the time being, too little is known on the structure-function of the *SPECC1L* product, on specific interactors and possible homo-dimerization properties. From the biological point of view instead, available findings have provided important indication on the cellular and physiological role of *SPECC1L*. The protein is a novel microtubule-actin cross-linking protein that interacts with components of the actin cytoskeleton and stabilizes microtubules, which is necessary for these fibers to regulate various cell processes including the migration of cells to their proper location and their cell–cell interactions mediated by adherens junctions [35,41]. Several model organisms showed that *SPECC1L* is particularly involved in the migration of cranial neural crest cells that will form craniofacial features [26,41,42]. Interestingly, different deletion mutants generated in the mouse show diverse degree of severity of defects further supporting the idea that position-specific mutations in the *SPECC1L* gene may account for different pathological phenotypes also in human [37].

Of note, the *MID1* protein, encoded by the gene responsible for the X-linked form of Opitz G/BBB syndrome, is also a microtubular protein [6,7,8] and the two products might physically and/or functionally interact contributing to the development of the embryonic midline structures. The patients reported here do not present mutations in the *MID1* coding exons and splice site consensi. The majority of them being males (26/30), we can rule out exon deletions due to hemizygosity of the gene. Further, many samples were molecularly karyotyped thus excluding the presence of large genomic abnormalities. However, future NGS analyses will be necessary to completely exclude the possibility of *MID1* involvement and to find the disease-causing gene(s). It is tempting to speculate that the *SPECC1L* variants identified in our work might exert a modifier effect on the primary pathogenetic cause whether it be *MID1* or other still unknown OS causing gene(s), thus contributing to the highly variable expressivity of the clinical signs [12,15,18].

## Figures and Tables

**Table 1:** Main clinical signs of this study patients.

Patient	Hypertelorsim and Other Facial Features	Laryngo–Tracheo–Esophageal Abnormalities	Hypospadias (Other External Genitalia Abnormalities)	Other Midline Signs ^a^
OS212	+		+	+
OS269 *	+		+	+
OS286	+	+	+	
OS293	+	+	+	
OS299	+		(+)	+
OS302	+		+	+
OS303	+		+	+
OS306	+		(+)	+
OS310 *	+		+	
OS311 (F)	+			+
OS312	+	+	+	
OS315			+	+
OS316 (F)	+			+
OS320 (F)	+	+		+
OS324	+	+	(+)	
OS325 *	+			+
OS326	+	+	+	+
OS327	+			
OS330	+	+		
OS331 *	+		+	+
OS332	+		+	+
OS336 *	+	+	+	+
OS337 (F)	+			+
OS338	+		+	+
OS345		+		+

^a^ One or more of other midline clinical signs reported, mainly: cleft of lip and/or palate, congenital heart and/or anal defects, cerebellar and/or corpus callosum hypoplasia. For OS296 *, OS305, OS334, OS335, OS346 info were not available. * Described below with *SPECC1L* variants detected.

**Table 2:** Missense variants identified in the *SPECC1L* gene.

Patient	Genomic Location ^a^	Exon ^b^	cDNA Alteration ^c^	Protein Alteration	dbSNP ^d^	Global MAF	SIFT	Mutation Taster	Polyphen2
OS310 OS325	22:24321542	5	c.562C>T	p.Leu188Phe	rs56168869	0.0034	Deleterious	Benign	Probably Damaging—score 0.998
OS296	22:24321580	5	c.600A>T	p.Leu200Phe	rs56112030	0.0094	Tolerated	Benign	Benign—score 0
OS331	22:24321669	5	c.689C>T	p.Thr230Ile	rs117220882	0.0058	Tolerated	Benign	Benign—score 0.002
OS269	22:24322440	5	c.1460G>A	p.Arg487His	rs55723436	0.0036	Tolerated	Benign	Probably Damaging—score 0.999
OS336	22:24328848	7	c.2149A>G	p.Thr717Ala	rs6004132	0.0006	Tolerated	Benign	Benign—score 0.001

^a^ Refseq_hg38; ^b^ numbering of coding exons; ^c^ cDNA numbering based on reference sequence GenBank NM_015330.4, 1 corresponds to the A of the ATG initiation translation codon; ^d^ from dbSNP build 155 (ncbi.nlm.nih.gov/snp, accessed on 30 November 2021).

**Table 3:** Frequencies of the *SPECC1L* variants identified.

Patient	cDNA Alteration	Protein Alteration	Global MAF	MAF GO- ESP	MAF ExAC	GnomAD	TOP Med	EAS	AMR	AFR	EUR	SAS
OS310 OS325	c.562C>T	p.Leu188Phe	0.0034	0.0057	0.0072	0.0085	0.005	0	0.011	0	0.009	0
OS296	c.600A>T	p.Leu200Phe	0.0094	0.0071	0.0116	0.0072	0.0056	0.002	0	0	0.013	0.033
OS331	c.689C>T	p.Thr230Ile	0.0058	-	0.0022	0.0023	0.0022	0.029	0	0	0	0
OS269	c.1460G>A	p.Arg487His	0.0036	0.0068	0.0057	0.0055	0.0058	0	0.006	0.001	0.01	0.003
OS336	c.2149A>G	p.Thr717Ala	0.0006	0.0015	0.0005	0.0012	0.0016	0	0	0.002	0	0

## Data Availability

The data presented in this study are available on request from the corresponding author. The data are not publicly available due to privacy restrictions.
